# Multistate transition modelling of e-cigarette use and cigarette smoking among youth in the UK

**DOI:** 10.1136/tc-2022-057777

**Published:** 2023-03-10

**Authors:** Jennie C Parnham, Charlotte Vrinten, Márta K Radó, Alex Bottle, Filippos T Filippidis, Anthony A Laverty

**Affiliations:** 1 Primary Care and Public Health, Imperial College London, London, UK; 2 Department of Management and Engineering, Institute for Analytical Sociology, Linköping University, Linköping, Sweden; 3 Department of Medical Epidemiology and Biostatistics, Karolinska Institutet, Stockholm, Sweden; 4 Dr Foster Unit, School of Public Health, Imperial College London, London, UK

**Keywords:** Electronic nicotine delivery devices, Co-substance use, Priority/special populations

## Abstract

**Introduction:**

E-cigarette use remains a controversial topic, with questions over how people transition between e-cigarette use and cigarette smoking. This paper examined transitions into and out of nicotine product use in a representative sample of UK youth.

**Methods:**

We used Markov multistate transition probability models on data from 10 229 participants (10–25 years old) in the UK Household Longitudinal Study (2015–2021). We used four product use states (‘never’, ‘non-current use’, ‘e-cigarette only’ and ‘smoking and dual use’) and estimated likelihood of transitions according to sociodemographic characteristics.

**Results:**

Among participants who had never used nicotine products, most were still non-users a year later (92.9% probability; 95% CI 92.6%, 93.2%); a small proportion transitioned to using e-cigarettes only (4.0%; 95% CI 3.7%, 4.2%) and cigarettes (2.2%; 95% CI 2.0%, 2.4%). Those aged 14–17 years were the most likely to start using a nicotine product. E-cigarette use was less persistent overtime than cigarette smoking, with a 59.1% probability (95% CI 56.9%, 61.0%) of e-cigarette users still using after 1 year compared with 73.8% (95% CI 72.1%, 75.4%) for cigarette smoking. However, there was a 14% probability (95% CI 12.8%, 16.2%) that e-cigarette users went onto smoke cigarettes after 1 year, rising to 25% (95% CI 23%, 27%) after 3 years.

**Conclusion:**

This study found that although overall nicotine product use was relatively rare, participants were more likely to experiment with e-cigarette use than cigarette smoking. This was mostly not persistent over time; however, approximately one in seven transitioned to cigarette smoking. Regulators should aim to deter all nicotine product use among children.

**WHAT IS ALREADY KNOWN ON THIS TOPIC**

- E-cigarettes remain controversial and there are concerns over both the extent of use among youth and whether e-cigarettes serve as a gateway to cigarette smoking.
- Markov transition models allow examination of complex transitions into and out of using different nicotine products, but so far, research using these methods has only been performed in the USA and for children 15 years old or older.
- The UK provides an important case study for e-cigarette policy as it has relatively high levels of use, a permissive regulatory regime and is governed by the European Union Tobacco Products Directive.

**WHAT THIS STUDY ADDS**

- This study applied a Markov transition model to a representative sample of UK children and youth (aged 10–25 years). It finds that being 14–17 years old and white were associated with taking up e-cigarettes and that non-users were more likely to take up e-cigarette use than cigarette smoking.
- However, e-cigarette use was found to be less persistent over time compared with cigarette smoking. Participants typically stayed smoking cigarettes for a longer period than participants remained using e-cigarettes. Approximately one in seven e-cigarette users transitioned to cigarette smoking.

**HOW THIS STUDY MIGHT AFFECT RESEARCH, PRACTICE OR POLICY**

- In these UK data, the majority of e-cigarette use among youth was experimentation. However, the greater likelihood of taking up e-cigarettes over cigarette smoking combined with the relatively high likelihood of transitioning between e-cigarette use and cigarette smoking highlights the need for regulation to deter any nicotine product use among children.

## Introduction

Electronic cigarettes (e-cigarettes) remain a controversial topic, with differences in legislation, regulation and popularity between countries.^1 2^ In the UK, an influential evidence synthesis by Public Health England (PHE) concluded that e-cigarettes are safer than tobacco smoking and may be effective for cessation.^3 4^ Conversely, other institutions, including WHO, have expressed concerns regarding the impact of e-cigarettes on health.^1^ Most commentators and the PHE report make a distinction between use by adults and use by children, with particular concern that e-cigarettes may provide a gateway into tobacco smoking for a new generation of people who smoke.^5^ Nicotine product use in adolescence is crucial to study as most nicotine product use in adulthood is initiated in adolescence.^6^ Surveys of 11–17 year-olds in Great Britain have found that 16% had tried e-cigarettes in 2022, a slight increase compared with 14% in 2020.^7^ Children from lower income households, from white ethnic backgrounds and from families where parents smoke are more likely to use e-cigarettes.^8 9^ Hence, if e-cigarettes are creating a new generation of people who smoke, this will contribute to tobacco-related health inequalities.^10^

Previous studies have examined the associations between e-cigarette use and tobacco uptake. The location, methodology and conclusions of these papers have varied. However, an association between e-cigarette use and tobacco uptake is supported by systematic reviews.^5 11^ A 2019 systematic review identified 17 studies on the relationship between e-cigarette use by non-smokers and later tobacco smoking.^11^ The review did find an association between e-cigarette use and smoking uptake, but heterogeneity was high, meaning that uncertainty persists. Some research has suggested a role for the addictive nature of nicotine in causing some people to move from e-cigarettes to tobacco, as in some cases tobacco can deliver nicotine more effectively.^11^ In contrast, it may be that common factors drive both e-cigarette and tobacco use, known as common liability.^12^ Based on systematic reviews, common liability is not well dealt with in the vast majority of research.^5 11 13^ The complex bidirectional relationship between nicotine product use needs further exploration.

More sophisticated analysis methods show promise in disentangling some of these issues. Research in the USA has modelled complex transitions into and out of e-cigarette and tobacco use using Markov models.^14–17^ These studies have commonly found that while e-cigarette use was a less persistent state than tobacco smoking over time,^14 16 17^ those who had used e-cigarettes were more likely to smoke tobacco compared with people who had never used e-cigarettes.^14–17^ Furthermore, these existing Markov models have only captured transitions among those aged 15 and over, so do not capture potential initial pathways into nicotine product use. These studies have been exclusively based in the USA, but the UK provides an important case study: the contents of e-cigarettes are controlled by the European Union Tobacco Products Directive, it has a relatively permissive regulatory system, and high levels of e-cigarette use.

Therefore, we aimed to examine the complex transitions between cigarette smoking and e-cigarette use in a representative sample of UK adolescents and young adults and to determine how the transitions are impacted by sociodemographic characteristics and change over time.

## Methods

### Data source

We used data from the UK Household Longitudinal Study (or ‘Understanding Society’), an annual longitudinal panel survey from 2009 onwards of over 28 000 households, representative of the UK population. Data on all household members are collected annually on socioeconomic, behavioural and emotional factors; a detailed survey methodology is described elsewhere.^18^ Household members were given an age-appropriate self-completion questionnaire. Participants aged 10–15 years and those over 16 years were given different questionnaires, with comparable questions on nicotine product use. We used data from 2015 to 2021 as questions on e-cigarette use were asked from 2015 onwards. To capture transitions between cigarette smoking and e-cigarette use in youth and early adulthood our analyses are of those aged 10–25 years old.

### Nicotine product use states

E-cigarette use was assessed with two questions that were included in the self-completion questionnaires. A single question ‘Do you ever use electronic cigarettes’ (response options: ‘yes’ and ‘no’) was asked to all respondents in wave 7 (2015/2016). From 2016 onwards (waves 8–12), participants aged 10–15 years were asked ‘Have you ever used e-cigarettes?’, while those who were 16 or older were asked, ‘Do you ever use e-cigarettes?’ (response options: ‘I have never’, ‘I have only tried once or twice’, ‘In the past but not now’, ‘Less than once a month’, ‘At least once a month but less than once a week’ and ‘At least once a week’). Participants were defined as ‘*e-cigarette users’* if they answered ‘yes’ in wave 7 or all responses except ‘never’ and ‘In the past but not now’ in waves 8–12.^19^

Cigarette smoking was assessed for all waves with the questions ‘Do you ever smoke cigarettes at all’ (ages 10–15 years) or ‘Do you smoke cigarettes?’ (ages 16–25 years). The response options were: ‘yes’ and ‘no’. Participants were defined as ‘*cigarette smoking*’ if they responded ‘yes’ to these questions.

We defined four states of nicotine product use for each wave. Participants were defined as ‘*never users’* if they reported having never used either e-cigarettes or tobacco cigarettes. Participants were defined as ‘*non-current users’* if they did not use nicotine products in that wave of data collection but had previously reported using a nicotine product. Due to the phrasing of the cigarette smoking question for 16–25 year-olds (‘Do you smoke cigarettes?’), some participants (n=800) were categorised as ‘*non-current users’* as their previous nicotine product could not be confirmed at their first wave of data collection. ‘*E-cigarette only users’* were those using only e-cigarettes in that wave. Participants were categorised as ‘*cigarette smoking*’ if they recorded smoking cigarettes in that wave. This group included those who only smoked cigarettes and those who were a ‘*dual user*’ of tobacco cigarettes and e-cigarettes. The ‘c*igarette smoking*’ and ‘*dual-use*’ states were combined due to low sample size and low levels of transition between the two states. However, as cigarette smoking is the most harmful nicotine product use state, we still capture the most important transitions for health.

### Covariates

The following covariates were considered in the analysis: age at the wave of data collection (categorised for analysis as 10–13, 14–17, 18–21 and 22–25 years); sex (male or female); ethnicity (white or ethnic minority); and tertiles of equivalised net monthly household income (low (<£1191/month), mid (£1191–£1787/month) and high (>£1787/month)). The Organisation for Economic Co-operation and Development equivalence scale was used to adjust household income by household composition.^20^ It was necessary to categorise continuous variables (age and income) for the use in the weighted Markov model function, ‘wmsm’,^14^ which at the time of analysis only accepted categorical covariates.

### Analyses

First, we present descriptive statistics for the characteristics of the sample for the first time point in the data (referred to as their baseline), stratified by their nicotine product use state at baseline.

#### Markov model

A continuous time multistate Markov model was then used to estimate the instantaneous probability of transitioning between the nicotine product use states.^21–23^ This model estimates the likelihood of transitioning based on the current state and the next state but not the previous states or the observation time (known as being ‘memoryless’). The transition probabilities that are estimated represent the collective likelihood of being in each state at a future point, based on the starting state.^14^ This model was chosen as it is flexible, allowing multiple transition pathways to occur unlike other time-to-event models which do not allow estimation of bidirectional transitions.^21 24^ Additionally, it optimised data use as all participants with two or more data points were included. Time-varying covariates, such as age, were accounted for using a piecewise constant model, whereby their value at each starting state was used.^23 25^

**Figure 1:**
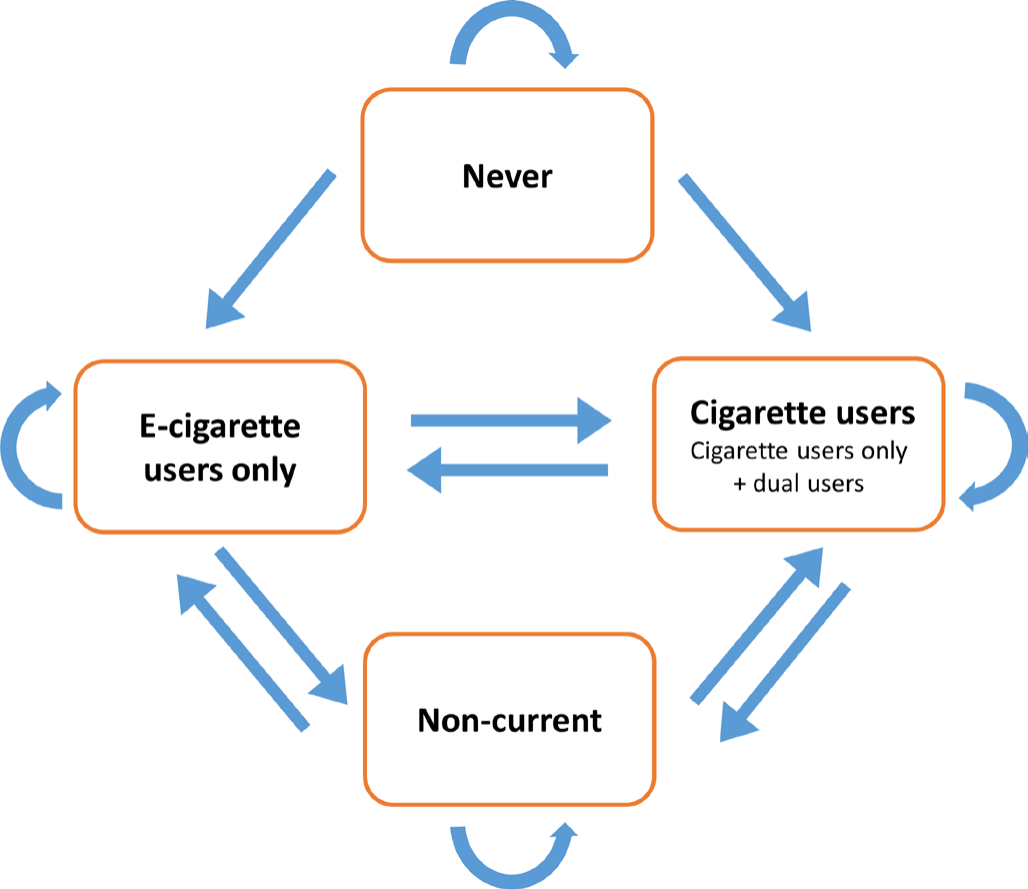
Permitted transitions between product use states in the Markov model. The cigarette users category includes both ‘dual-use’ and ‘cigarette use only’ states.

Permitted transitions between the nicotine product use states are described in figure 1. Transitions overtime were predicted through multiplying the transition matrix, with each iteration corresponding to one year. The model was adjusted for survey weights using the ‘wmsm’ function developed by Brouwer *et al*.^14^ To determine whether transition probabilities were affected by sociodemographic characteristics, the model was adjusted for age, sex, ethnicity and household income.

### Sensitivity analyses

A property of Markov models is that they are ‘time-homogenous’ and do not depend on the observation year. However, as there is evidence that uptake of e-cigarettes during the study period increased,^26^ this assumption may not hold. To test the assumption, transition intensities were estimated for two-wave subsamples of the data and compared with the total sample. We found that transition intensities for wave 7 (2015–2016) had a wider CI than other waves and the total sample. This is likely due to the change in measurement of e-cigarette use between waves 7 and 8. Therefore, sensitivity analyses were conducted excluding wave 7 from the analysis. Furthermore, we tested the impact of COVID-19 by excluding any waves of data collection that occurred while COVID-19 restrictions were in place in the UK (March 2020 to July 2021). To test the impact of categorising participants over the age of 16 with no smoking history data as non-current users (n=800), we excluded these participants and reran the model.

## Results

### Study participants

There were 14 919 participants aged 10–25 years in the data set between 2015 and 2021, of which 10 478 (70%) had participated in more than one wave. Participants were additionally excluded if they had missing nicotine product use information (n=170) or missing covariate information (ethnicity: n=25; income: n=54). The final analytical sample size was 10 229, of which 2447 (23%) were followed for two waves and 7782 (77%) were followed for three or more waves (table 1).

**Table 1:** Baseline characteristics for 10 229 participants aged 10–25 years in the UK Household Longitudinal Study (2015–2021) stratified by their baseline nicotine product use state

Characteristic		Cigarette smoking (n=911, 9%)	E-cigarette user only (n=337, 3%)	Non-current user (n=1708, 17%)	Never (n=7273, 71%)	Total n=10 229	P value*
Number of waves, n (%)							<0.001
2		289 (32)	109 (32)	490 (29)	1502 (21)	2390 (23)	
3		223 (24)	90 (27)	412 (24)	1457 (20)	2182 (21)	
4		178 (20)	53 (16)	342 (20)	1400 (19)	1973 (19)	
5		135 (15)	58 (17)	245 (14)	1401 (19)	1839 (18)	
6		86 (9.4)	27 (8.0)	219 (13)	1513 (21)	1845 (18)	
Age at baseline, n (%)							<0.001
10–13		38 (4.2)	30 (8.9)	40 (2.3)	3540 (49)	3648 (36)	
14–17		194 (21)	115 (34)	396 (23)	1941 (27)	2646 (26)	
18–21		351 (39)	89 (26)	663 (39)	1185 (16)	2288 (22)	
22–25		328 (36)	103 (31)	609 (36)	607 (8.3)	1647 (16)	
Sex, n (%)							<0.001
Male		448 (49)	207 (61)	764 (45)	3411 (47)	4830 (47)	
Female		463 (51)	130 (39)	944 (55)	3862 (53)	5399 (53)	
Ethnicity, n (%)							<0.001
White		758 (83)	265 (79)	1048 (61)	5218 (72)	7289 (71)	
Ethnic minority		153 (17)	72 (21)	660 (39)	2055 (28)	2940 (29)	<0.001
Household income, n (%)							
Low		402 (44)	123 (36)	699 (41)	2725 (37)	3949 (39)	
Middle		289 (32)	118 (35)	526 (31)	2503 (34)	3436 (34)	
High		220 (24)	96 (28)	483 (28)	2045 (28)	2844 (28)	

Cigarette smoking includes both ‘dual-users’ and ‘cigarette users only’ states. Values are unweighted.

*Pearson’s χ^2^ test for difference in covariates across nicotine product use state.

Of the total sample, 7273 (71%) were never users at baseline, 37 (3%) used e-cigarettes only, 991 (9%) were cigarette users (including dual use and cigarette smoking only) and 1708 (17%) were non-current users. Compared with never users at baseline, participants who used e-cigarettes or smoked cigarettes at baseline were more likely to be older and of white ethnicity. E-cigarette users were more likely to be male than cigarette users or non-current users, and cigarette users were more likely to be in the low income category than e-cigarette users at baseline.

### One-year transition probabilities

**Figure 2:**
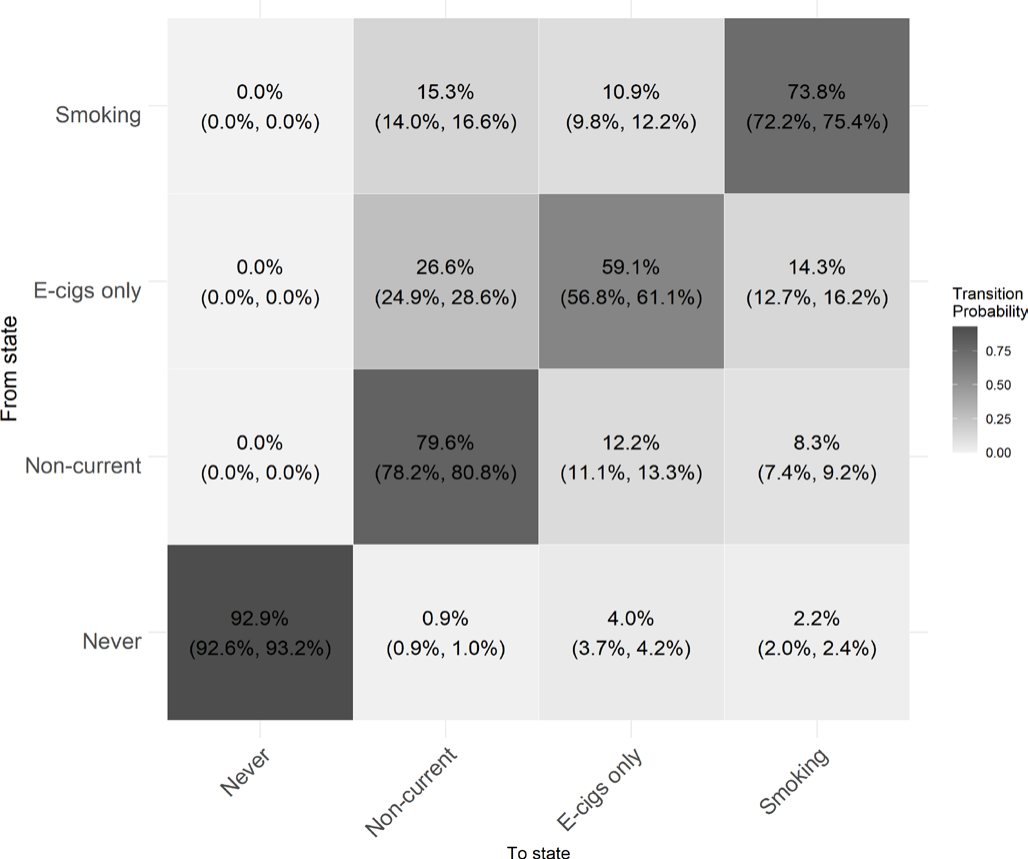
One-year unadjusted transition probabilities showing the likelihood of transitioning between four nicotine product use states in a sample of British 10–25 year-olds. Smoking includes ‘dual-use’ and ‘cigarette smoking only’ states. E-cigs, electronic cigarettes.

The one-year transition probabilities show the likelihood of transitioning from one state to another in the next year (figure 2).

Among participants who had never used nicotine products, there was a 92.9% probability that they were still never users a year later (95% CI 92.6%, 93.2%), a 4.0% probability that they transitioned to e-cigarette use (95% CI 3.7%, 4.2%) and a 2.2% probability that they transitioned to cigarette use (95% CI 2.0%, 2.4%). Among participants who were non-current nicotine product users, there was a 79.6% probability that they would remain non-users a year later (95% CI 78.1%, 80.8%). Non-current users were more likely to transition to e-cigarette use (12.2%; 95% CI 11.2%, 13.3%) than cigarette smoking (8.3%; 95% CI 7.4%, 9.3%). Among participants who used e-cigarettes, there was a 59.1% probability that they remained e-cigarette users a year later (95% CI 56.9%, 61.0%). E-cigarette users were more likely to transition into non-current use (26.6%; 95% CI 24.8%, 28.6%) than into cigarette smoking (14.3%; 95% CI 12.8%, 16.2%) after 1 year. Among participants who smoked cigarettes, there was a 73.8% probability of remaining a cigarette user after one year (95% CI 72.1%, 75.4%). Of those cigarette users who transitioned, there was a slightly higher likelihood of transitioning to non-current use (15.3%; 95% CI 14.0%, 16.8%) than e-cigarette use (10.9%; 95% CI 9.7%, 12.1%).

### Impact of sociodemographic characteristics on transition probabilities

**Figure 3:**
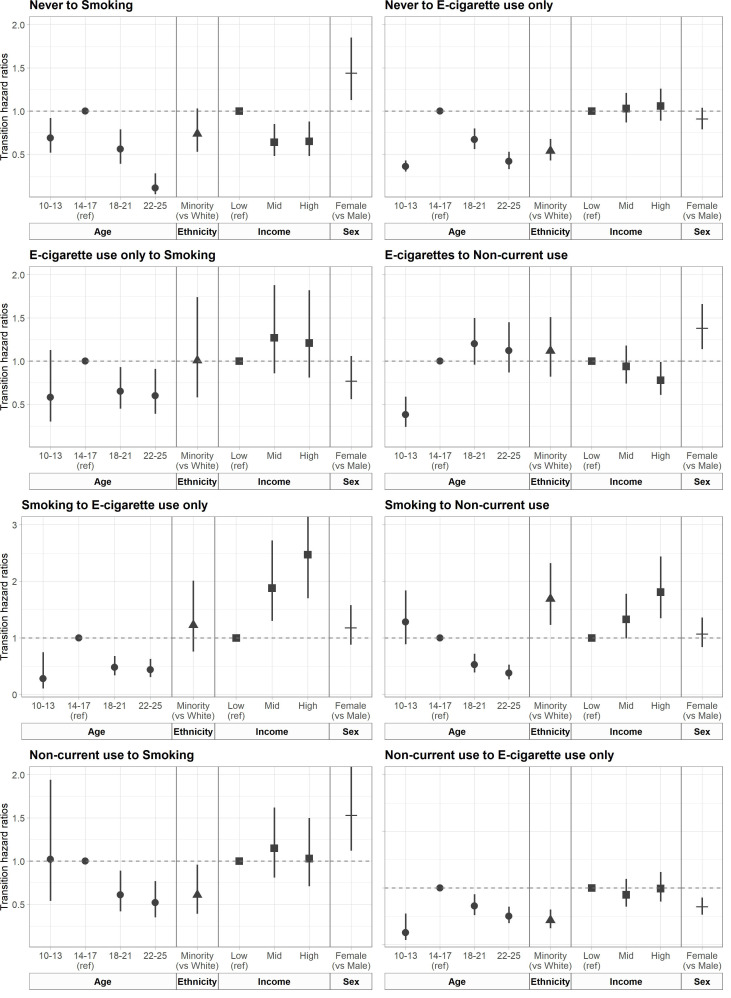
HRs showing the relative differences in the likelihood of transitioning between four nicotine product use states by age, ethnicity, income and sex in a sample of British 10–25 year-olds. Smoking includes ‘dual-use’ and ‘cigarette smoking only’ states. Numerical values are displayed in online supplemental table 1. E-cigarette, electronic cigarette.

We examined the impacts of covariates on transition probabilities, with relative effects estimated as HRs (figure 3) (online supplemental table 1). Participants who had never used nicotine products were more likely to transition into smoking if they were aged 14–17 years or female but were less likely to transition into smoking if they were high income (vs low income). Never users were more likely to transition into e-cigarette use if they were 14–17 year-olds than all other age groups but less likely to transition into e-cigarettes if they were from an ethnic minority (vs white).

10.1136/tc-2022-057777.supp1 Supplementary data

Among participants who were non-current users, they were less likely to transition to cigarette smoking if they were older (18–21 and 22–25 years vs 14–17 years), from an ethnic minority (vs white) and male (vs female).

Among participants who used e-cigarettes, they were less likely to transition to non-current use if they were younger (10–13 vs 14–17 years), high income (vs low income) or male (vs female). E-cigarette users were less likely to transition into cigarette smoking if they were older (18–21 and 22–25 years vs 14–17 years).

Participants who smoked cigarettes were more likely to transition to e-cigarette use if they were 14–17 years compared with other age groups and high or middle income compared with low income. Participants who smoked cigarettes were less likely to transition to non-current use if they were older (18–21 and 22–25 years vs 14–17 years) but more likely to transition if they were high income (vs low income) or of an ethnic minority (vs white).

### Transition probabilities over time

**Figure 4:**
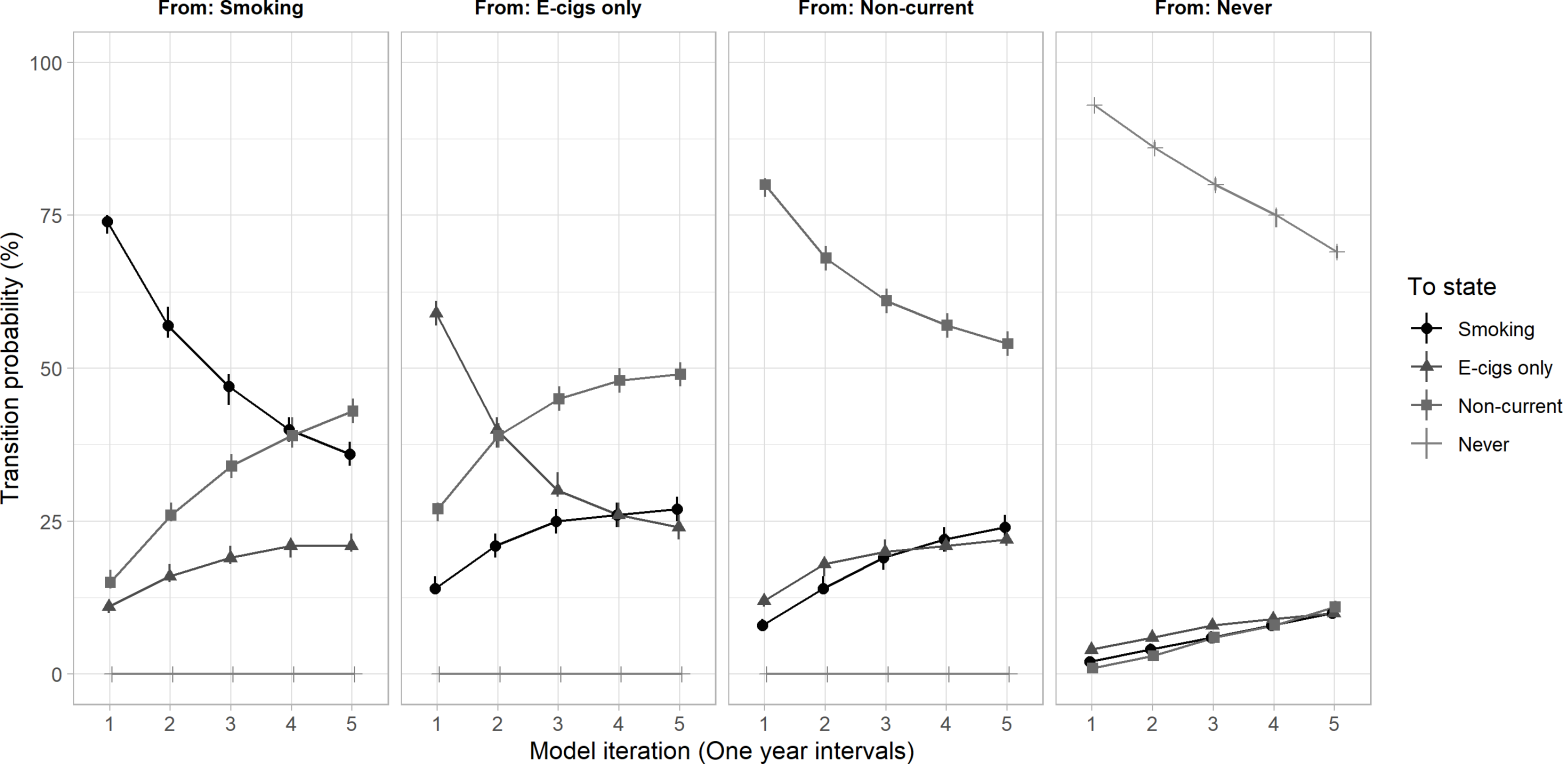
Transition probabilities showing the predicted likelihood of transitioning between four nicotine product use states estimated over five model iterations (equivalent to 5 years) in a sample of British 10–25 year-olds. Smoking includes ‘dual-use’ and ‘cigarette smoking only’ states. Numerical values are displayed in online supplemental table 2. E-cigs, electronic cigarettes.

Among those who were never users, there was a 69% probability that they were still non-users after five years (95% CI 68%, 70%) (figure 4). There was a higher probability of never users transitioning to e-cigarette use than cigarette smoking, which increased for the first three years (e-cigarettes year 3: 8%, 95% CI 8%, 9%; cigarette smoking year 3: 6%, 95% CI 6%, 7%). However, after year three, there was a similar likelihood of never users transitioning to e-cigarettes or cigarette smoking (year 5: 10%, respectively).

Among e-cigarette users the probability of remaining an e-cigarette user fell over time (year 1: 59%; year 5: 25%) while the likelihood of transitioning to non-current use increased (year 1: 27%; year 5: 46%). After two years, the likelihood of e-cigarette users transitioning to non-current use was greater than that of remaining an e-cigarette user. The likelihood of e-cigarette users transitioning to cigarette smoking rose over time from 14% in year one to 27% in year five.

Among participants who smoked cigarettes, the probability of remaining a cigarette user fell over time (year 1: 74%; year 5: 37%) while the likelihood of transitioning to non-current use increased (year 1: 15%; year 5: 41%). However, the likelihood of transitioning to non-current use was only higher than remaining a cigarette user after five years. Overtime, the likelihood that cigarette users transitioned to using e-cigarettes rose from 11% in year one to 22% in year five.

### Sensitivity analyses

Our results were robust to sensitivity analyses. Although estimates were found to not be homogenous over time, being different in wave 7 compared with later waves (online supplemental figure 1), repeating the analyses excluding wave 7 produced similar results (online supplemental figure 2). Furthermore, we tested the impact that COVID-19 restrictions may have had on transition probabilities by excluding any data points which occurred between March 2020 and July 2021. We found that estimates were similar in the models which included and did not include COVID-19 years, as the CIs overlapped for all transitions (online supplemental figure 3). To test the impact of categorising participants over the age of 16 with no smoking history data as non-current users, we excluded these participants and reran the analysis (online supplemental figure 4). We found that estimates were similar in both samples, suggesting that our findings were robust to any potential misclassification bias.

## Discussion

We analysed a representative sample of 10 229 adolescents and young adults in the UK over six waves of data collection, finding that those who had never or did not currently use a nicotine product were more likely to take up e-cigarettes only than cigarette smoking. Overtime, e-cigarette users were more likely to become non-users than those who smoked cigarettes. This suggests that most e-cigarette use was experimentation and that those who started cigarette smoking were more likely to continue smoking for a longer period than e-cigarette users. Mid-adolescence (14–17 years) was the period when most started using nicotine products.

Our findings indicate that adolescents were more likely to experiment with e-cigarette use than cigarette smoking, especially at ages 14–17 years. This is consistent with the literature. Brouwer *et al* 14 and Niaura *et al*,^16^ who used Markov models to explore transitions between nicotine product states in US adults, similarly found evidence that e-cigarette use was the least persistent state over time compared with cigarette smoking and non-use states. Perceptions over the social acceptability of e-cigarette use could explain this finding. Research has suggested that adolescents view experimental e-cigarette use relatively positively compared with regular e-cigarette use or cigarette smoking,^27^ and that trying different flavours or products could be driving e-cigarette experimentation compared with cigarette smoking.^27 28^

We found that participants who smoked cigarettes were more likely to remain using the same product after 5 years compared with e-cigarette users. We also found that older participants were less likely to transition away from cigarette smoking. While evidence for the gateway effect from this study is unclear, there was a relatively high likelihood of transitioning between nicotine product use states which rose over time, suggesting a bidirectional relationship, possibly explained through common liability.^5 11 13^ It is notable that the likelihood that e-cigarette users transitioned into cigarette smoking was significantly higher than never users transitioning into cigarette smoking. Considering this bidirectional relationship between e-cigarette use and smoking, it is concerning that the likelihood of transitioning away from never use rises over time and throughout adolescence.

### Strengths and limitations

This study used a large, nationally representative sample of the UK, with recent data to accurately reflect the state of e-cigarette use in adolescents today. The age range from 10 to 25 years, unlike previous research on this topic, allowed for examination of the initial transitions into nicotine product use. The use of multistate Markov models enabled us to capture complex bidirectional pathways into and out of nicotine product use.

There are some limitations to note. Our sample size was limited as the e-cigarette question was only included in the survey from 2015 (wave 7) onwards. Furthermore, participants aged 16 years and over were asked about current not ever use of cigarettes. As a result, some participants aged 16+ at their baseline measurement with no previous data were assumed to be non-current users, if they reported not using nicotine products (n=800). After sensitivity analyses excluding these participants, however, our results were found to be robust to any potential misclassification bias. Due to the low prevalence of transitions, we had to combine ‘*dual-use*’ with ‘*cigarette smoking only’*. Therefore, some nuances of the relationship could be obscured, but overall, the most important transitions for health were captured. Also, the nicotine product use states did not necessarily reflect the intensity of use. Those merely trying nicotine products were combined with regular users due to the difference in questions across years (binary in wave 7 and multicategorical from wave 8). Nicotine product use was self-reported and so could be misreported. However, previous studies have validated self-reported e-cigarette use and cigarette smoking with data from biomarkers.^29^ Furthermore, only a limited number of covariates could be added to the model as the statistical power needed for the Markov model to converge was high.

## Conclusions

This study highlights that adolescents were more likely to experiment with e-cigarettes than cigarette smoking and that the uptake of all nicotine products was greatest between ages 14 and 17 years. To prevent the establishment of nicotine product use in later life, these findings show the need for regulation to deter any nicotine product use among children. These findings are especially critical considering the recent trend of increasing e-cigarette use in UK adolescents.

## Data Availability

Data are available in a public, open-access repository (UK data service) under End User Licence conditions. The data can be accessed at https://doi.org/10.5255/UKDA-SN-6614-16.
