# Supplementary material for: Multistate transition modelling of e-cigarette use and cigarette smoking among youth in the UK
Source: Tob Control. 2023 Mar 10;33(4):489–96. doi: 10.1136/tc-2022-057777 (PMC11228222; doi:10.1136/tc-2022-057777)
Supplement: Supplementary data [file tc-2022-057777supp001.pdf]

SUPPLEMENTARY FILES

List of Supplementary Tables and Figures

Supplementary Table 1 - Hazard ratios showing the relative differences in the likelihood of transitioning from three nicotine-product use states by age, ethnicity, income, and sex. 2
Supplementary Table 2 - Adjusted transition probabilities showing the likelihood of transitioning between five nicotine-product use state estimated over four model iterations (equivalent to four years). 3

Supplementary Figure 1 – Comparison of transition probabilities by observation year (two-wave subsection) to test the time homogeneity of transition probabilities... 4
Supplementary Figure 2 - Comparison of transition probabilities from the main analyses (six waves of data, waves 7-12) and the sensitivity analyses that excluded wave 7 (four waves of data, waves 8-12)... 4
Supplementary Figure 3 – Comparison of transition probabilities from the main analyses (all six waves of data) and the sensitivity analyses that excluded transitions that occurred during COVID-19 restrictions (March 2020 – July 2021) 5
Supplementary Figure 4 – Comparison of transition probabilities from the main analyses (all six waves of data) and the sensitivity analyses that excluded participants whose baseline nicotine product use status was assumed to be 'non-current' (n=800). 5

Supplementary Table 1 - Hazard ratios showing the relative differences in the likelihood of transitioning from three nicotine-product use states by age, ethnicity, income, and sex.

Covariate	From: Never		From: E-cigarettes		From: Smoking		From: Non-current	
	To: e-cigs	To: smoking	To: non-current	To: smoking	To: non-current	To: e-cigs	To: e-cigs	To: smoking
	HR (95% CI)	HR (95% CI)	HR (95% CI)	HR (95% CI)	HR (95% CI)	HR (95% CI)		
Age (years)								
10-13	0.36 (0.3, 0.43)	0.69 (0.52, 0.92)	0.38 (0.24, 0.59)	0.58 (0.3, 1.13)	1.28 (0.89, 1.84)	0.28 (0.11, 0.75)	0.21 (0.08, 0.55)	1.02 (0.54, 1.94)
14-17 (ref)	1.00	1.00	1.00	1.00	1.00	1.00	1.00	1.00
18-21	0.67 (0.56, 0.8)	0.56 (0.39, 0.79)	1.2 (0.96, 1.5)	0.65 (0.45, 0.93)	0.53 (0.39, 0.72)	0.48 (0.34, 0.68)	0.68 (0.52, 0.89)	0.61 (0.42, 0.89)
22-25	0.42 (0.33, 0.53)	0.11 (0.04, 0.28)	1.12 (0.87, 1.45)	0.6 (0.39, 0.91)	0.38 (0.27, 0.53)	0.44 (0.31, 0.63)	0.5 (0.38, 0.67)	0.52 (0.35, 0.77)
Ethnicity								
Minority (vs White)	0.54 (0.43, 0.68)	0.74 (0.53, 1.03)	1.12 (0.82, 1.51)	1.01 (0.58, 1.74)	1.69 (1.23, 2.32)	1.23 (0.76, 2.01)	0.43 (0.29, 0.62)	0.61 (0.39, 0.96)
Income								
Low (ref)	1.00	1.00	1.00	1.00	1.00	1.00	1.00	1.00
Mid	1.03 (0.87, 1.21)	0.64 (0.48, 0.85)	0.94 (0.74, 1.18)	1.27 (0.86, 1.88)	1.33 (0.99, 1.78)	1.88 (1.3, 2.72)	0.88 (0.67, 1.16)	1.15 (0.81, 1.62)
High	1.06 (0.89, 1.26)	0.65 (0.48, 0.88)	0.78 (0.61, 0.99)	1.21 (0.81, 1.82)	1.81 (1.35, 2.44)	2.47 (1.7, 3.6)	0.99 (0.76, 1.28)	1.03 (0.71, 1.5)
Sex								
Female (vs Male)	0.91 (0.79, 1.04)	1.44 (1.13, 1.85)	1.38 (1.14, 1.66)	0.77 (0.56, 1.06)	1.07 (0.84, 1.36)	1.18 (0.88, 1.58)	0.67 (0.53, 0.83)	1.53 (1.12, 2.11)

Supplementary Table 2 - Adjusted transition probabilities showing the likelihood of transitioning between five nicotine-product use state estimated over four model iterations (equivalent to four years).

	**Year 1**	**Year 2**	**Year 3**	**Year 4**	**Year 5**
	% (95% CI)	% (95% CI)	% (95% CI)	% (95% CI)	% (95% CI)
**From: Smoking**					
To: Smoking	74 (72, 75)	57 (55, 59)	47 (44, 49)	40 (38, 42)	36 (34, 38)
To: E-cigarettes only	11 (10, 12)	16 (15, 18)	19 (18, 21)	21 (19, 22)	21 (20, 23)
To: Non-current	15 (14, 17)	26 (25, 28)	34 (32, 36)	39 (37, 42)	43 (41, 45)
To: Never	0 (0, 0)	0 (0, 0)	0 (0, 0)	0 (0, 0)	0 (0, 0)
**From: E-cigarettes only**					
To: Smoking	14 (13, 16)	21 (19, 23)	25 (23, 27)	26 (24, 28)	27 (25, 29)
To: E-cigarettes only	59 (57, 61)	40 (37, 42)	30 (28, 32)	26 (24, 28)	24 (23, 26)
To: Non-current	27 (25, 29)	39 (37, 41)	45 (43, 47)	48 (46, 50)	49 (47, 51)
To: Never	0 (0, 0)	0 (0, 0)	0 (0, 0)	0 (0, 0)	0 (0, 0)
**From: Non-current**					
To: Smoking	8 (7, 9)	14 (13, 16)	19 (17, 20)	22 (20, 24)	24 (22, 26)
To: E-cigarettes only	12 (11, 13)	18 (16, 19)	20 (19, 22)	21 (20, 23)	22 (20, 24)
To: Non-current	80 (78, 81)	68 (66, 70)	61 (59, 63)	57 (55, 59)	54 (52, 56)
To: Never	0 (0, 0)	0 (0, 0)	0 (0, 0)	0 (0, 0)	0 (0, 0)
**From: Never**					
To: Smoking	2 (2, 2)	4 (4, 5)	6 (6, 7)	8 (7, 9)	10 (9, 10)
To: E-cigarettes only	4 (4, 4)	6 (6, 7)	8 (8, 9)	9 (9, 10)	10 (10, 11)
To: Non-current	1 (1, 1)	3 (3, 3)	6 (5, 6)	8 (8, 9)	11 (10, 12)
To: Never	93 (93, 93)	86 (86, 87)	80 (79, 81)	75 (73, 76)	69 (68, 71)

Note: Smoking includes both 'dual-use' and' cigarette only' states.

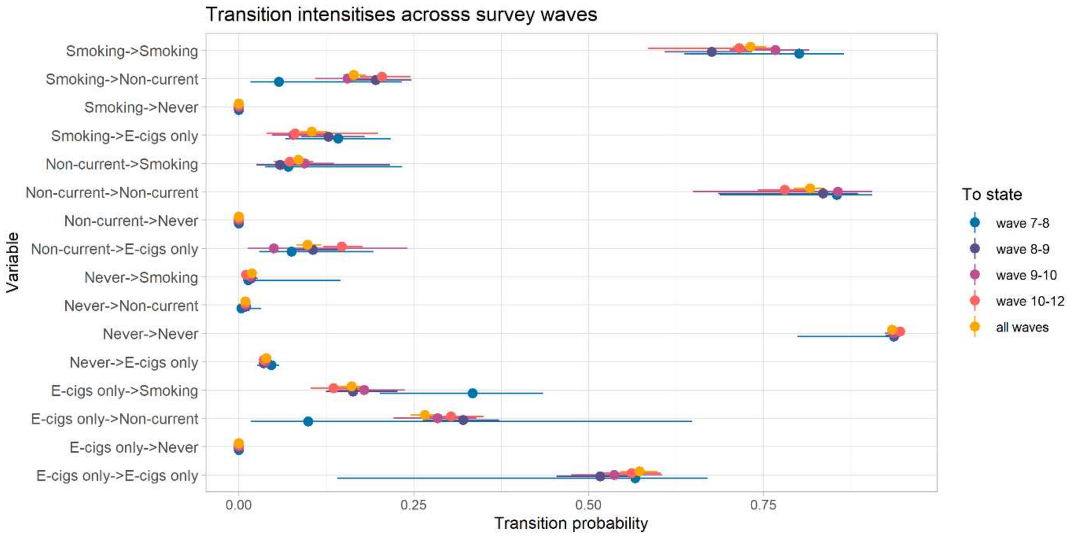

Supplementary Figure 1 – Comparison of transition probabilities by observation year (two-wave subsection) to test the time homogeneity of transition probabilities

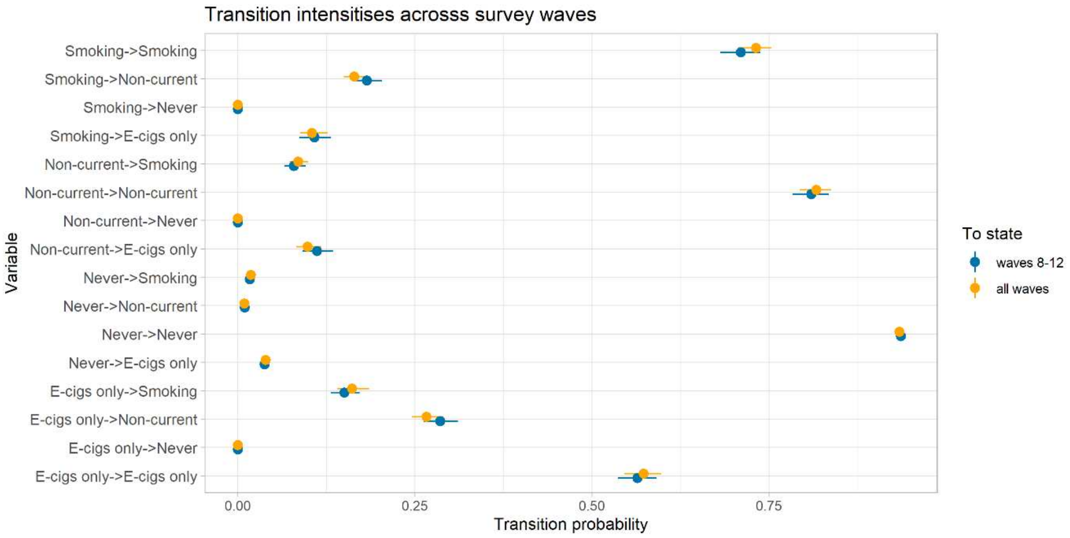

Supplementary Figure 2 - Comparison of transition probabilities from the main analyses (six waves of data, waves 7-12) and the sensitivity analyses that excluded wave 7 (four waves of data, waves 8-12)

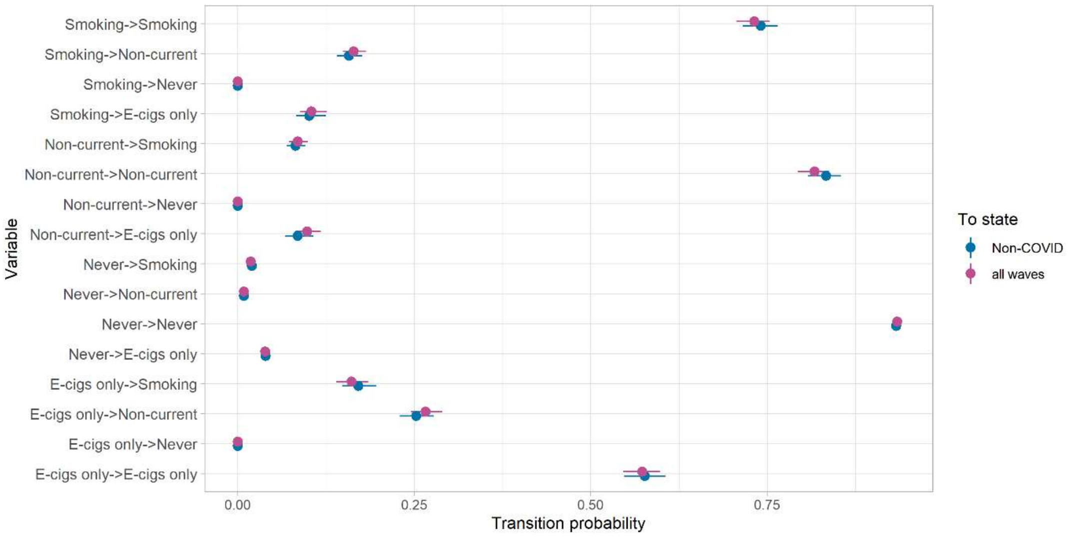

Supplementary Figure 3 – Comparison of transition probabilities from the main analyses (all six waves of data) and the sensitivity analyses that excluded transitions that occurred during COVID-19 restrictions (March 2020 – July 2021)

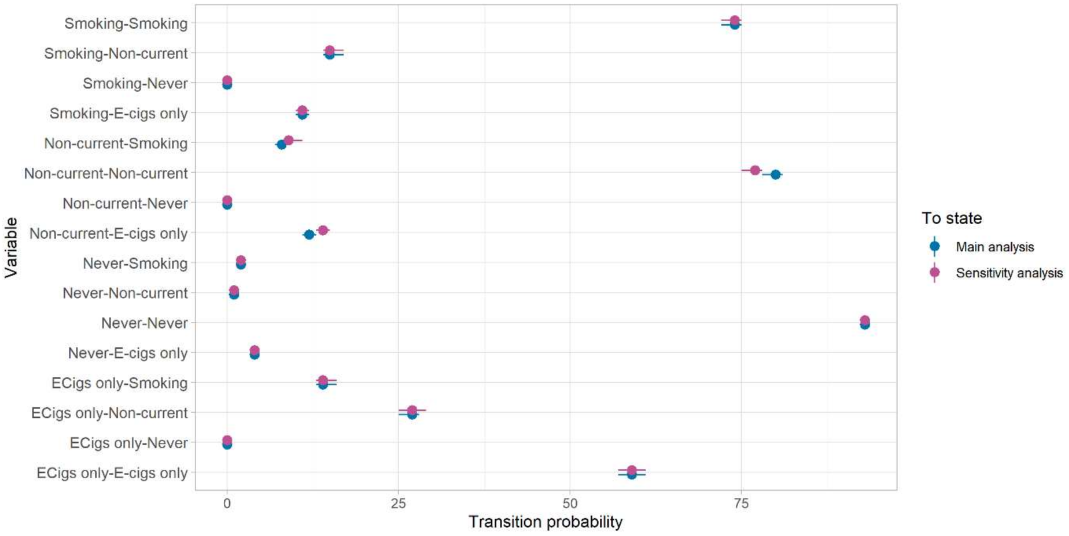

Supplementary Figure 4 – Comparison of transition probabilities from the main analyses (all six waves of data) and the sensitivity analyses that excluded participants whose baseline nicotine product use status was assumed to be 'non-current' (n=800).
